# A Germline Clone Screen on the *X* Chromosome Reveals Novel Meiotic Mutants in *Drosophila melanogaster*

**DOI:** 10.1534/g3.112.003723

**Published:** 2012-11-01

**Authors:** Kimberly A. Collins, Jonathon G. Callicoat, Cathleen M. Lake, Cailey M. McClurken, Kathryn P. Kohl, R. Scott Hawley

**Affiliations:** * Stowers Institute for Medical Research, Kansas City, Missouri 64110; † Department of Biological Sciences, University of Missouri, Kansas City, Missouri 64110; ‡ Curriculum in Genetics and Molecular Biology, University of North Carolina, Chapel Hill, North Carolina 27599; § Department of Molecular and Integrative Physiology, University of Kansas Medical Center, Kansas City, Kansas 66160

**Keywords:** meiosis, mutant screens, chromosome segregation

## Abstract

In an effort to isolate novel meiotic mutants that are severely defective in chromosome segregation and/or exchange, we employed a germline clone screen of the *X* chromosome of *Drosophila melanogaster*. We screened over 120,000 EMS-mutagenized chromosomes and isolated 19 mutants, which comprised nine complementation groups. Four of these complementation groups mapped to known meiotic genes, including *mei-217*, *mei-218*, *mei-9*, and *nod*. Importantly, we have identified two novel complementation groups with strong meiotic phenotypes, as assayed by *X* chromosome nondisjunction. One complementation group is defined by three alleles, and the second novel complementation group is defined by a single allele. All 19 mutants are homozygous viable, fertile, and fully recessive. Of the 9 mutants that have been molecularly characterized, 5 are canonical EMS-induced transitions, and the remaining 4 are transversions. In sum, we have identified two new genes that are defined by novel meiotic mutants, in addition to isolating new alleles of *mei-217*, *mei-218*, *mei-9*, and *nod*.

A cornerstone of investigating Drosophila female meiosis has been the power and success of genetic screens. In the first EMS mutagenesis screen for meiotic mutants in flies, Baker and Carpenter (1972) screened 209 mutagenized *X* chromosomes and isolated six strong meiotic mutants, whose further investigation proved to be a critical foundation for studies of the mechanisms of meiotic chromosome segregation and recombination (Hawley 1993). For example, the Baker and Carpenter (1972) screen identified *nod*, *mei-218*, *mei-9*, *mei-38*, *mei-41*, and *Klp3A^mei-352^*, among other mutants. Since the landmark Baker and Carpenter screen, two additional screens for *X*-linked meiotic mutants have been performed. Sekelsky *et al.* (1999) screened 2311 mutagenized *X* chromosomes using *P* element mutagenesis and identified an essential meiotic regulator, *mei-P26*. Most recently, Liu *et al.* (2000) screened 2106 EMS-mutagenized *X* chromosomes and identified *mei-217* and *hdm*.

Similarly, traditional screening of either wild populations and/or EMS-mutagenized chromosomes have proved fruitful in their identification of genes such as *ord*, *mei-S332*, *mei-S282*, *mei-P22*, *CycE*, *mei-W68*, *mei-S51*, and *sub* (Giunta *et al.* 2002; Mason 1976; Sandler 1971; Sandler *et al.* 1968; Sekelsky *et al.* 1999). Taken together, the characterization of the mutants resulting from these screens has greatly contributed to our current understanding of fundamental processes in Drosophila female meiosis, including recombination, cohesion, achiasmate chromosome segregation, and meiotic spindle organization.

Most recently, Page *et al.* (2007) advanced the art of genetic screens in Drosophila female meiosis when they performed a germline clone screen to identify autosomal meiotic mutants. This screen created germline clones in females heterozygous for the mutagenized autosome. In this screen, the use of dominant female sterile mutation *ovo^D1-18^* on the un-mutagenized homolog ensured that the only fertile progeny resulted from germline clones homozygous for the EMS-mutagenized chromosome. Meiotic mutants were selected by crossing these females to a compound autosome, thus demanding that the female nondisjoin an autosome in order to have viable progeny.

This strategy has three salient advantages: it is in essence an F1 screen; it is a selection and not a screen for mutants; and it has the ability to isolate lethal mutants. Using this technique, Page *et al.* (2007) isolated 11 new meiotic mutants on 2*L* and 3*R* in a screen of 46,388 EMS-mutagenized chromosomes. Importantly, this screen identified three novel meiotic mutants: *cona*, *mcm5*, and *trem* (Page *et al.* 2007), whose subsequent characterization has illuminated meiotic chromosome synapsis (*cona*), a requirement for *mcm5* in the resolution of crossovers and the mechanism by which double-strand breaks are initiated in meiosis (*trem*) (Lake and Hawley 2012; Lake *et al.* 2007, 2011; Page *et al.* 2008).

In a continuation of the germline clone approach for meiotic mutant isolation, we sought to identify novel fertile meiotic mutants on the *X* chromosome with strong effects on either chromosome segregation or recombination. A limitation of this strategy is that the germline clone-bearing female must undergo high levels of nondisjunction and be reasonably fertile. Prior to our screen, the notable meiotic mutations on the *X* included *mei-217*, *mei-218*, *mei-9*, *hdm*, *mei-P26*, *mei-41*, *mei-38*, *east*, *Cap*, *Klp3A^mei-352^*, and *nod*. As alleles of *mei-217*, *mei-218*, *mei-9*, and *hdm* are fertile, we anticipated isolating new alleles of these genes. As strong hypomorphic and null alleles of *mei-P26*, *mei-41*, *Klp3A^mei-352^*, and *Cap* have greatly reduced fertility or are sterile, we did not anticipate isolating strongly hypomorphic alleles of these genes. Finally, as *nod* primarily affects the achiasmate chromosome segregation pathway, we did not expect to isolate any alleles of this gene (Carpenter 1973).

Here we describe the isolation of 19 novel meiotic mutants on the *X* chromosome using the approach pioneered by Page *et al.* (2007). Among the 19 mutants, we isolated nine complementation groups, of which four correspond to *mei-217*, *mei-218*, *mei-9*, and *nod*. Three of the unidentified complementation groups isolated demonstrate only weak to moderate levels of meiotic nondisjunction and will not be pursued further. The final two complementation groups represent novel genes. Mutants in both of these complementation groups display strong chromosome nondisjunction phenotypes, and the characterization of these mutants will be described elsewhere.

We molecularly characterized the lesions in *mei-217*, *mei-218*, *mei-9*, and *nod* and were able to identify mutations in 9 of the 10 mutants within the coding regions. Of these lesions, six were nonsense mutations, and three were missense mutations. Only five of the mutations were traditional EMS-induced transitions. In sum, we have identified two novel meiotic mutants in addition to isolating new alleles of *mei-217*, *mei-218*, *mei-9*, and *nod*.

## Materials and Methods

### Drosophila stocks

All stocks were maintained on standard medium containing yeast, cornmeal, corn syrup, malt extract, and agar at 25° with the exception of the stocks containing *P{hs-hid}Y*, which were maintained at room temperature.

### Stocks used in the screen

*y^1^ w^1118^ FRT19A/P{hs-hid}Y* derived from Bloomington 1744 *y^1^ w^1118^ P{ry[+t7.2]=neoFRT}19A*
*P{ovoD1-18}P4.1*, *P{hsp70-flp}1*, *y^1^ w^1118^ sn^3^ P{neoFRT}19A/ P{hs-hid}Y* derived from Bloomington 23880 *P{ovoD1-18}P4.1*, *P{hsp70-flp}1*, *y^1^ w^1118^ sn^3^ P{neoFRT}19A/C(1)DX*, *y^1^ w^1^ f^1^*
*C(2)EN*, *b^1^pr^1^* (Bloomington 1112)
*C(1)DX*, *y^1^ f^1^/FM7i /Y* (Bloomington 5263)
FM7a/y+Y
*X^Y*, *In(1)EN,v f B*; *C(4)RM,ci ey^R^ x C(1)RM,y v*; *C(4)RM*, *ci ey^R^*
*w^1118^ PBac{WH}mei-217^f04441^ mei-218^f04441^* (Bloomington 18770)
*y mei-218^1^ / C(1)DX*, *y f / y^+^Y*; *spa^pol^*
*Dp(1;1)sc^V1^*, *y^1^ mei-218^1^ car^1^*, *y^+^/C(1)DX*, *y^1^ f^1^* (Bloomington 4914)
*y hdm^g7^/C(1)DX*, *y f/y^+^Y*; *spa^pol^* (K. S. McKim)
*w^1^ mei-9^A2^/C(1)DX*, *y^1^ f^1^* (Bloomington 4280)
*y nod^a^/C(1)DX*, *y^1^ f^1^ /y^+^Y* ; *spa^pol^*
*Df(1)BSC719*, *P+PBac{XP3.WH3}BSC719 w^1118^/FM7h/Dp(2;Y)G*, *P{hs-hid}Y*(Bloomington 26571; Df(*mei-38*))
*Df(1)BSC537*, *w^1118^/FM7h/Dp(2;Y)G*, *P{hs-hid}Y* (Bloomington 25065; Df(*mei-P26*))
*Df(1)BSC760*, *w^1118^ P+PBac{XP3.WH3}BSC760/Binsinscy* (Bloomington 26857; Df(*Cap*))
*Df(1)ED7364*, *w^1118^ P{3′.RS5+3.3′}ED7364/FM7h* (Bloomington 9905; Df(*mei-41*))
*Df(1)ED6565*, *P{3′.RS5+3.3′}ED6565 w^1118^/FM7h* (Bloomington 9299; Df(*east*))
*Df(1)ED411*, *P{3′.RS5+3.3′}ED411 w^1118^/FM7j*, *B1* (Bloomington 8031; Df(*Klp3A^mei-352^*))

### Determination of lethal hit rate

To determine the lethal hit rate induced by 35 mM EMS mutagenesis in females, 200 vials of the following cross were analyzed for the presence of Bar and non-Bar male progeny: y^1^ w^1118^P{ry[+t7.2]=neoFRT}19A**/*FM7a*; *spa^pol^*/*+* crossed to y^1^ w^1118^P{ry[+t7.2]=neoFRT}19A**/*y^+^Y*; *spa^pol^* /+, where asterisks (**) indicate the mutagenized chromosome. Ten of 195 vials had exclusively Bar male progeny and therefore represent a lethal hit on the *X* chromosome. As 35 mM EMS was used for all rounds of mutagenesis, this represents a 5.1% lethal hit rate in the screen. The 35 mM concentration of EMS was chosen because higher doses of EMS did not result in an increased rate of male lethality in the assay described above.

### Germline clone screen genetics

*y^1^ w^1118^ P{ry[+t7.2]=neoFRT}19A* / *P{hs-hid}Y* stock bottles were heat shocked for one hour at 38° on day 5 after egg laying, and the resulting virgin females were collected for EMS mutagenesis. Mutagenized virgins were mated to *P{ovoD1-18}P4.1*, *P{hsp70-flp}1*, *y^1^ w^1118^ sn^3^ P{neoFRT}19A*/ *P{hs-hid}Y* males in bottles. The parents were brooded into new bottles at day 3 and allowed to lay for three additional days before bottles were cleared. For both broods, the larvae were heat shocked at days 3, 4, and 5 for one hour at 38° to induce germline clone formation via mitotic recombination and to induce expression of *hid*. The resulting virgin females containing germline clones of the genotype *y^1^ w^1118^ P{ry[+t7.2]=neoFRT}19A / P{ovoD1-18}P4.1*, *P{hsp70-flp}1*, *y^1^ w^1118^ sn^3^ P{neoFRT}19A* were crossed to *C(2)EN*, *b^1^ pr^1^* males. The *C(2)EN*, *b^1^ pr^1^* crosses were tested with 1, 3, or 10 virgins in a vial, as well as 25 or 30 females in a bottle. In all, six rounds of EMS mutagenesis were performed, and the ideal culture conditions were determined to be 10 virgins in a vial. Vials or bottles were screened initially on day 10 for the presence of pupae. All vials or bottles with zero or one pupa were discarded at day 10. Progeny from the remaining vials were collected until day 18. Males or females that were not bearing *C(2)EN b^1^ pr^1^* were isolated for stock establishment. Male progeny were genotypically y^1^ w^1118^ ** P{ry[+t7.2]=neoFRT}19A / *Y* and were crossed as single males to *C(1)DX*, *y^1^ f^1^* /*Y* virgin females to establish stocks. The mutant is indicated by (**). Female progeny were y^1^w^1118^ ** P{ry[+t7.2]=neoFRT}19A / *+* and were mated singly with *FM7i* / *Y* males. From this cross, y^1^ w^1118^ **? P{ry[+t7.2]=neoFRT}19A? / *FM7i* virgin females were again crossed to *FM7i* / *Y* males for stock establishment. The “*?*” indicates unknown presence of the mutant (**) and the *FRT* site in the line. Once stocks were established, females were tested for the presence of the *FRT* site by PCR. Homozygous y^1^ w^1118^ **? P{ry[+t7.2]=neoFRT}19A mutant chromosomes were then retested for *X* and *4^th^* chromosome nondisjunction. Similarly, male stocks were made homozygous (y^1^ w^1118^ ** P{ry[+t7.2]=neoFRT}19A) and were then retested for *X* and *4^th^* chromosome nondisjunction. Of the stocks that were initially isolated from females, all were able to be maintained in the male as *y^1^ w^1118^ mutant P{ry[+t7.2]=neoFRT}19A* / *C(1)DX*, *y^1^ f^1^* /*Y* stocks.

### EMS mutagenesis

Virgin females (75 females per bottle, 20 bottles) were starved for six hours in bottles lacking medium. While females were starving, empty bottles with four Whatman #3 circular filter papers were securely taped to the bottom of empty 8 oz round-bottom fly bottles. Three milliliters of 35 mM EMS in 3% sucrose was pipetted into each bottle containing the Whatman filter paper. EMS was allowed to absorb fully before adding starved virgins at a density of 75 virgins/bottle. Flies were allowed to ingest EMS for 24 hr, and then the flies were transferred to bottles containing normal fly food medium for 24 hr. Next, 100 males were transferred into fresh food bottles, and the mutagenized females were added to these bottles. Progeny were reared at 25°.

### Heat-shock procedure

Heat-shock treatment of bottles was performed as previously published (Page *et al.* 2007) for round 1 of the screen, but for rounds 2–6, the heat shock was done on days three, four, and five, as it was experimentally determined that heat shocking larvae on these three consecutive days yielded the maximal number of germline clone-containing progeny, as assayed by *sn^3^* mosacism in the *y^1^ w^1118^ P{ry[+t7.2]=neoFRT}19A / P{ovoD1-18}P4.1*, *P{hsp70-flp}1*, *y^1^ w^1118^ sn^3^ P{neoFRT}19A* females.

### Screening for *FRT19A* in mutants recovered from females

Single fly squashes were performed on aged females according to Gloor *et al.* (1993), and the resulting DNA was assayed for the presence of the *FRT* site using primers 5′*cgcagatataggtgcgacgtg*3′ and 5′*gccgtatgggtctacttgacag*3′, which yielded a PCR product of 403 bp when the *FRT* site was present.

### Complementation testing and assays for chromosome nondisjunction

Complementation was assayed within the 19 mutants by crossing transheterozygote virgins to *X^Y*, *In(1)EN,v f B*; *C(4)RM,ci ey^R^* males and assaying *X* and *4^th^* chromosome nondisjunction by methods reported previously (Hawley *et al.* 1993; Zitron and Hawley 1989). All of the mutations isolated are fully recessive.

For complementation testing of the mutants against known meiotic mutants on the *X* chromosome, either deficiency stocks or mutant alleles for *mei-217*, *mei-218*, *mei-38*, *east*, *Klp3A^mei-352^*, *mei-P26*, *mei-41*, *Cap*, *mei-9*, and *nod* were used. Due to the inconsistent levels of nondisjunction observed in homozygotes of the three weakest complementation groups, we were unable to determine whether any of these three groups represent new alleles of *hdm*. Transheterozygotes were tested for complementation by crossing to *y sc cv v f car* / *B[S]Y* males; *spa^pol^* males and assaying *X* chromosome nondisjunction (Matsubayashi and Yamamoto 2003; Zimmering 1976).

### Sequencing

DNA was isolated from a single aged male according to Gloor *et al.* (1993). Sequencing primers for *mei-217*, *mei-218*, *mei-9*, and *nod* are available upon request.

### Metaphase I oocytes preparations and microscopy

DAPI-only preparations of metaphase I oocytes and microscopy was performed as previously described (Gilliland *et al.* 2009).

### Saturation calculations

The number of alleles per mutable locus (*m*) is calculated as the number of alleles divided by the number of loci. Percentage saturation is calculated as 100 (1 − *e*^−^*^m^*) (Laurencon *et al.* 2004).

## Results and Discussion

To identify novel fertile meiotic mutants on the *X* chromosome of *Drosophila melanogaster*, we undertook a large-scale screen employing a FLP-FRT–mediated germline clone strategy that is analogous to the strategy used in screens for meiotic mutants on 2*L* and 3*R* by the Hawley Laboratory (Page *et al.* 2007). While at least three screens for meiotic mutants on the *X* chromosome have been performed, we suspected that additional fertile meiotic mutants were yet to be discovered, as only 4626 mutagenized *X* chromosomes had been screened (Baker and Carpenter 1972; Liu *et al.* 2000; Sekelsky *et al.* 1999). Similar to the screens performed by Page and colleagues, we utilized a dominant female sterile mutation *ovo^D^* (*P{ovoD1-18}P4.1)* in combination with the creation of germline clones such that the only fertile offspring following germline clone induction are due to FLP-induced recombinants that lack *ovo^D^* and are therefore homozygous for the *y^1^ w^1118^ P{ry[+t7.2]=neoFRT}19A* chromosome.

**Figure 1:**
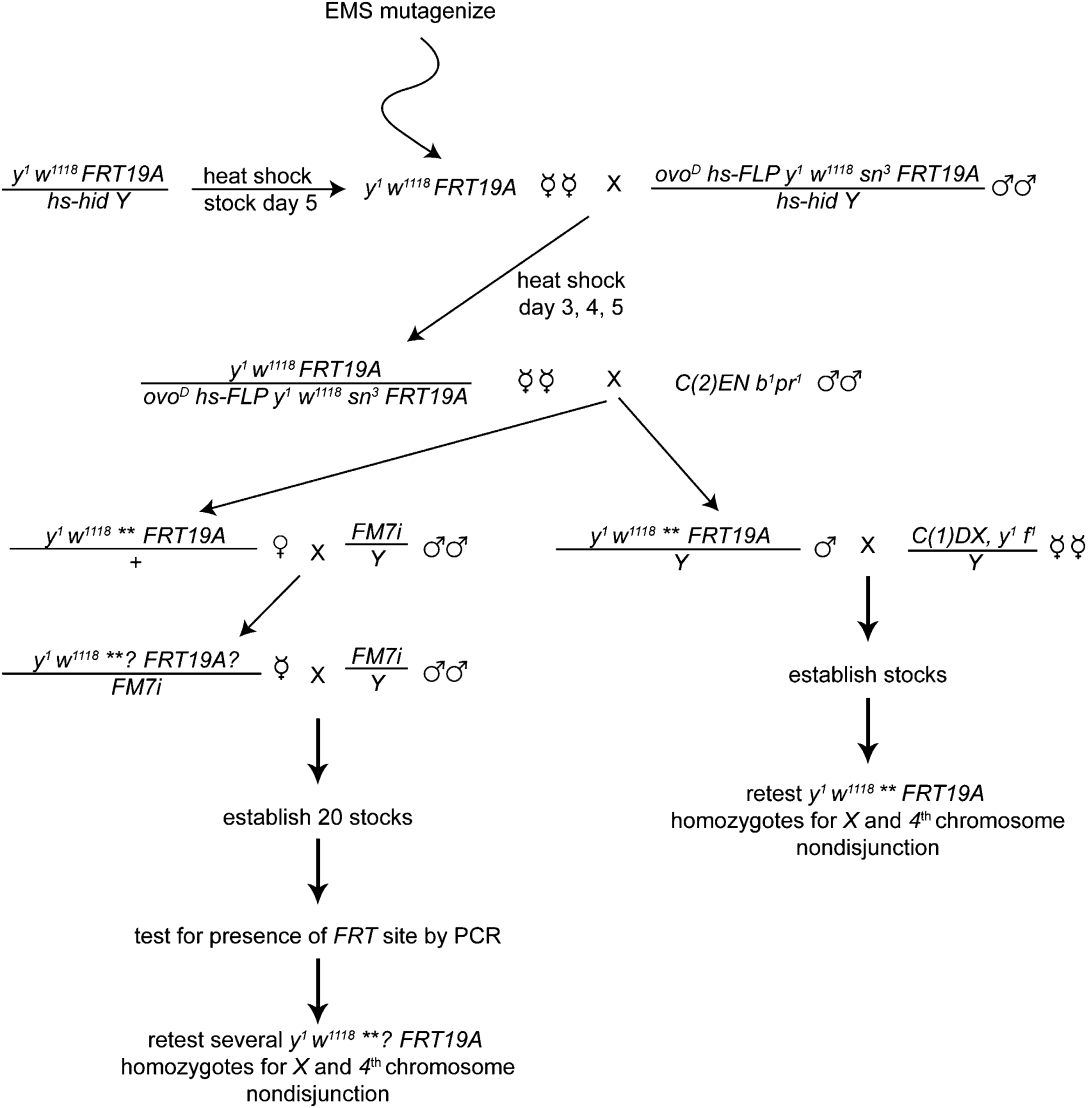
Schematic representing the cross schemes used in the screen to isolate new meiotic mutants on the *X* chromosome. Specifics of the screen are detailed in *Materials and Methods*.

We performed EMS mutagenesis on *y^1^ w^1118^ P{ry[+t7.2]=neoFRT}19A* females and then crossed them to *P{ovoD1-18}P4.1*, *P{hsp70-flp}1*, *y^1^ w^1118^ sn^3^ P{neoFRT}19A*/*P{hs-hid}Y* males (Figure 1). We then crossed the resulting germline clone containing female progeny of the genotype *y^1^ w^1118^ P{ry[+t7.2]=neoFRT}19A/ P{ovoD1-18}P4.1*, *P{hsp70-flp}1*, *y^1^ w^1118^ sn^3^ P{neoFRT}19A* to males bearing a compound second chromosome, *C(2)EN b^1^ pr^1^* (Figure 1).

This cross selects for meiotic mutants because progeny only arise following nondisjunction of the second chromosome in the female [see Page *et al.* (2007)] (Figure 1A). Nondisjunctional progeny arise from the combination of an *X*; diplo *2* egg and a *Y*; nullo *2* sperm or from an *X*; diplo *2* egg and an *X*; nullo *2* sperm. Although *C(2)EN b^1^ pr^1^* progeny will also arise, these were discarded because the presence of the compound autosome prohibited further analysis of the mutant. Mutant stocks were preferentially established from males by crossing a single male to *C(1)DX*, *y^1^ f^1^*/*Y* females (Figure 1). When available, males were chosen for the stock establishment because establishing stocks from a female required an extra generation and a PCR screening step to identify stocks with the *FRT* site (Figure 1).

In six rounds of EMS mutagenesis, 121,048 *X* chromosomes were screened. Any vial or bottle with more than one pupa was kept for the chromosome recovery step (see *Materials and Methods*). In all, 77 putative mutants were isolated, with the majority (63.6%) being from vials with two or three progeny. All 77 stocks were retested for *X* and *4^th^* chromosome nondisjunction by crossing homozygous mutant females to *X^Y*, *In(1)EN,v f B*; *C(4)RM,ci ey^R^* males. Of the 77 stocks, 19 mutants (24.7%) showed elevated levels of *X* and/or *4^th^* chromosome nondisjunction, and all are homozygous viable (Table 1 and data not shown). Surprisingly, the number of exceptions per vial was not a strong indicator of whether the vial contained a meiotic mutant (data not shown).

**Table 1:** Nondisjunction frequencies in 19 novel meiotic mutants

Gamete Type											
Maternal	Paternal	*y w*	*mei-39^1^*	*mei-39^166^*	*mei-39^129^*	*mei-826*	*mei-217^1330^*	*mei-218^125^*	*mei-218^621^*	*mei-218^1940^*	*mei-218^136^*
*X*; *4*	*XY*; *44*	366	62	95	121	88	141	52	44	80	78
*X*; *4*	*0*; *44*	442	99	135	140	137	222	39	75	110	108
*X* ND											
*0*; *4*	*XY*; *44*	1	20	33	22	30	25	14	10	15	17
*XX*; *4*	*0*; *44*	2	32	33	37	45	48	13	15	26	31
*4* ND											
*X*; *0*	*XY*; *44*	1	21	22	12	22	18	7	8	9	11
*X*; *0*	*0*; *44*	3	27	22	28	37	30	1	6	26	8
*X*; *44*	*XY*; *0*	2	8	11	24	18	18	11	14	11	6
*X*; *44*	*0*; *0*	0	13	16	18	22	20	2	8	12	10
*X*; *4* ND											
*0*; *0*	*XY*; *44*	0	17	45	30	14	15	6	2	22	17
*XX*; *44*	*0*; *0*	0	8	18	11	16	11	2	2	5	14
*0*; *44*	*XY*; *0*	0	3	0	1	4	2	5	0	0	3
*XX*; *0*	*0*; *44*	0	4	8	6	4	6	0	1	7	4
**Total progeny**		**808**	**314**	**438**	**450**	**437**	**556**	**152**	**185**	**323**	**307**
Adjusted total		820	398	575	557	550	663	192	215	398	393
% *X* nondisjunction		0.7	42.2	47.7	38.4	41.1	32.3	41.7	27.9	37.7	43.8
% *4* nondisjunction		0.7	33.4	37	32	31.8	23.2	24.5	21.4	31.7	28.2
% nullo-*X*		0.24	20.1	27.1	19.0	17.5	12.7	26.0	11.2	18.6	18.8
% diplo-*X*		0.49	22.1	20.5	19.4	23.6	19.6	15.6	16.7	19.1	24.9
% nullo-*4*		0.49	22.6	26.1	20.1	17.3	13.6	10.4	9.3	23.4	15.5
% diplo-*4*		0.24	10.8	11.0	11.8	14.5	9.7	14.1	12.1	8.3	12.7

Gamete Type											
Maternal	Paternal	*mei-218^646^*	*mei-218^1057^*	*mei-9^140^*	*mei-9^357^*	*nod^143^*	*mei-105*	*mei-175*	*mei-86*	*mei-114*	*mei-889*
*X*; *4*	*XY*; *44*	129	145	101	175	89	170	357	476	32	255
*X*; *4*	*0*; *44*	173	161	106	229	104	257	558	645	90	324
*X* ND											
*0*; *4*	*XY*; *44*	26	31	10	12	6	16	7	28	4	5
*XX*; *4*	*0*; *44*	42	40	14	36	3	24	3	6	3	4
*4* ND											
*X*; *0*	*XY*; *44*	11	18	9	13	355	14	17	14	2	20
*X*; *0*	*0*; *44*	19	23	13	17	555	11	20	24	8	30
*X*; *44*	*XY*; *0*	23	12	13	23	15	11	11	24	7	28
*X*; *44*	*0*; *0*	18	13	9	28	18	15	13	21	13	17
*X*; *4* ND											
*0*; *0*	*XY*; *44*	19	21	5	3	12	10	2	0	0	0
*XX*; *44*	*0*; *0*	12	10	5	10	0	5	0	0	0	0
*0*; *44*	*XY*; *0*	3	5	3	5	0	1	0	2	0	2
*XX*; *0*	*0*; *44*	10	9	3	2	7	3	0	6	1	0
**Total progeny**		**485**	**488**	**291**	**553**	**1164**	**537**	**988**	**1246**	**160**	**685**
Adjusted total		597	604	331	621	1192	596	1000	1288	168	696
% *X* nondisjunction		37.5	38.4	24.2	21.9	4.7	19.8	2.4	6.5	9.5	3.2
% *4* nondisjunction		26.6	25.8	23	19.5	82.3	14.9	6.5	7.7	19	14.2
% nullo-*X*		16.1	18.9	10.9	6.4	3.0	9.1	1.8	4.7	4.8	2.0
% diplo-*X*		21.4	19.5	13.3	15.5	1.7	10.7	0.6	1.9	4.8	1.1
% nullo-*4*		14.7	16.7	11.5	6.4	79.5	8.6	4.1	3.9	7.1	7.2
% diplo-*4*		11.9	9.1	11.5	13.0	2.8	6.4	2.4	3.8	11.9	7.0

All mutants are homozygous viable and were tested as y^1^ w^1118^^*^^*^ P{ry[+t7.2]=neoFRT}19A ; *spa^pol^* homozygote females crossed to *X^Y*, *In(1)EN,v f B*; *C(4)RM,ci ey^R^* males to assay *X* and *4^th^* chromosome nondisjunction (where ^*^^*^ indicates the mutation). In all cases, 25 females were analyzed.

We placed the mutants into complementation groups by testing transheterozygotes for *X* chromosome nondisjunction. We found nine complementation groups (data not shown), of which four had multiple alleles. Next, we tested representative members of each complementation group against known meiotic mutants on the *X* chromosome using deficiency stocks and/or mutant alleles of *mei-217*, *mei-218*, *east*, *mei-P26*, *mei-41*, *Cap*, *mei-9*, *mei-38*, *Klp3A^mei-352^*, and *nod* (data not shown). We found that the largest complementation group represents six new alleles of *mei-218* (which we name *mei-218^125^*, *mei-218^136^*, *mei-218^621^*, *mei-218^1057^*, *mei-218^646^*, and *mei-218^1940^*). In addition, another complementation group identified two novel alleles of *mei-9* (*mei-9^357^* and *mei-9^140^*). This analysis also identified mutant line *143* as an allele of *nod* (*nod^143^*) and mutant line *1330* as an allele of *mei-217 (mei-217^1330^)*.

Strikingly, the two complementation groups with the strongest nondisjunction phenotype complemented all meiotic mutants tested and therefore represent two novel complementation groups. The first group is composed of three alleles, and we have preliminarily named mutant line *39* (*mei-39^1^*), mutant line *129* (*mei-39^129^*), and mutant line *166* (*mei-39^166^*). The second novel complementation group is composed of a single allele preliminarily named *mei-826*.

Complementation analysis of one of the groups (*mei-114*, *mei-86*, and *mei-175*) was not completed, as these homozygotes did not have a reproducible nondisjunction phenotype in this assay. Therefore, we were unable to ascertain whether *mei-114*, *mei-889*, and *mei-105* are alleles of known meiotic genes. However, it was clear that mutant lines *mei-114*, *mei-889*, and *mei-105* failed to complement one another in all combinations.

One meiotic mutant that was not included in the initial complementation testing is *hdm*, as all strong complementation groups were accounted for by *mei-217*, *mei-218*, *mei-9*, and *nod*, and the two novel complementation groups that show the highest level of nondisjunction do not map to *hdm* (data not shown). It is possible that one of the weak complementation groups (represented by *mei-114*, *mei-889*, and *mei-105*) are allelic to *hdm*; however, results were inconclusive due to low levels of nondisjunction in *hdm* homozygotes and due to variable nondisjunction frequencies in the *mei-114*, *mei-889*, and *mei-105* homozygotes. Characterization of the two novel complementation groups (represented by *mei-39^1^* and *mei-826*) will be described in subsequent articles.

To accurately assay *4^th^* chromosome nondisjunction in our mutant stocks, we crossed y^1^ w^1118^ ** P{ry[+t7.2]=neoFRT}19A; *spa^pol^* females to *X^Y*, *In(1)EN,v f B*; *C(4)RM,ci ey^R^* males and scored all resulting progeny (Table 1). Mutants within a complementation group exhibited similar frequencies of nondisjunction.

Eleven mutants demonstrated *X* chromosome nondisjunction at 25% or above. Among these 11 mutants, 5 showed 40% or greater *X* chromosome nondisjunction (*mei-39^1^*, *mei-39^166^*, *mei-826*, *mei-218^125^*, and *mei-218^136^*) and 6 showed *X* chromosome nondisjunction between 25 and 40% (*mei-217^1330^*, *mei-218^621^*, *mei-218^1940^*, *mei-218^646^*, *mei-218^1057^*, and *mei-39^129^*). The remaining 8 mutant stocks exhibited weaker *X* chromosome nondisjunction phenotypes, ranging between 2.4 and 24.2% (*mei-9^140^*, *mei-9^357^*, *nod^143^*, and *mei-105*, *mei-175*, *mei-86*, *mei-114*, and *mei-889*). All mutants displayed an approximately equal ratio of nullo-*X* to diplo-*X* eggs, although mutant lines *mei-39^1^*, *mei-39^166^*, *mei-39^129^*, and *mei-218^1940^* yielded about twice as many nullo-*4* eggs to diplo-*4* eggs, potentially indicating a *4^th^* chromosome loss phenotype (Table 1).

To molecularly characterize the lesions in *mei-217*, *mei-218*, *mei-9*, and *nod*, we sequenced the exons of all alleles in the respective complementation groups and compared them with the parental *y^1^ w^1118^ P{ry[+t7.2]=neoFRT}19A* sequence. In 9 of 10 mutants sequenced, we identified lesions in the coding sequence (Table 2). In 6 of 10 cases, the mutation encoded a nonsense mutation, whereas three cases were missense mutations. We were unable to identify any mutations in the exons of *mei-218^1057^*. This may be due to the fact that we did not obtain high-quality sequence reads for a small region in exons 6 and 7. Alternatively, the mutation in *mei-218^1057^* may lie in the regulatory regions.

**Table 2:** Mutations identified in novel meiotic mutants

Allele	Mutation	Canonical	Amino Acid Change
*mei-217^1330^*	A to T	No	K111 ter
*mei-218^125^*	C to T	Yes	Q339 ter
*mei-218^621^*	A to T	No	K320 ter
*mei-218^136^*	G to A	Yes	W365 ter
*mei-218^646^*	C to T	Yes	Q1014 ter
*mei-218^1940^*	T to A	No	S845R
*mei-218^1057^*	Unknown	Unknown	Unknown
*mei-9^140^*	G to A	Yes	G930I
*mei-9^357^*	A to T	No	K408 ter
*nod^143^*	T to C	Yes	I620T

Exons of *mei-217*, *mei-218*, *mei-9*, and *nod* were sequenced for the mutants that failed to complement *mei-217*, *mei-218*, *mei-9*, or *nod*, respectively. All mutations were identified within coding regions with the exception of *mei-218^1057^*, for which no mutation was identified. The *mei-218^1057^* lesion may be in noncoding or regulatory regions. Alternatively, the mutation could be within one of two gaps (176 bp in total) of exon 6 and exon 7 for which we were unable to obtain high quality sequence in *mei-218^1057^*.

Of the nine mutants that we molecularly characterized, five were canonical EMS-induced transitions, and four were non-traditional transversions. Unlike the previous germline clone screen for meiotic mutants (Page *et al.* 2007), we did not obtain mutants due to mobilization of Doc elements. Note that we will make all recovered alleles fully available for at least one year, but we may not maintain the weaker mutants past one year.

### Isolation of novel meiotic mutants in a germline clone screen

In our screen of 121,048 mutagenized *X* chromosomes, all 19 novel meiotic mutants that we recovered are homozygous viable. Because our screen could have identified homozygous lethal mutants, it is surprising that we did not create a null mutation in an essential gene that yielded a meiotic phenotype. When we also consider that the Page *et al*. (2007) germline clone screen found only 2 of 11 mutants to be homozygous lethal, together, both screens isolated only 2 of 30 (6.7%) homozygous lethal mutants. Therefore, neither screen yielded a substantial number of meiotic mutants that were recessive lethal. Perhaps our dearth of homozygous-lethal mutants is related to a serious limitation of the screen: an inability to obtain maternal-effect lethals.

Among the nine complementation groups we identified, four represented previously characterized genes (*mei-217*, *mei-218*, *mei-9*, and *nod*). It is our hope that the new alleles may help shed light on the understanding of their respective gene functions. For example, Nod is a chromokinesin-like protein that functions in achiasmate (or nonexchange) chromosome segregation. *In vitro*, Nod can stimulate microtubule assembly and is responsible for the polar ejection force that maintains achiasmate chromosomes on the meiotic spindle (Cui *et al.* 2005; Theurkauf and Hawley 1992). Indeed, live imaging of *nod* null oocytes reveals that achiasmate *X* and *4^th^* chromosomes are rapidly ejected from the spindle shortly following spindle assembly (Hughes *et al.* 2009; Theurkauf and Hawley 1992). Our novel allele, *nod^143^*, has a nondisjunction phenotype that is nearly identical to the null allele *nod^a^* (Carpenter 1973). Interestingly, the mutation occurs at amino acid 620, which is the same amino acid in which the complex rearrangement in *nod^b17^* begins (Rasooly *et al.* 1994) (Table 2). *Nod^143^* represents the only allele of *nod* isolated to date that is a genetic null and a single missense mutation, as *nod^a^* truncates the last 12 amino acids of the protein, and all other genetically null alleles are the result of complex rearrangements or contain deletions (Rasooly *et al.* 1994). Because Nod functions primarily to ensure that achiasmate *X* and *4^th^* chromosomes properly disjoin at meiosis I, we did not anticipate the isolation of an allele of *nod* in a screen for autosomal nondisjunction mutants. Retrospectively, we note that Carpenter (1973) showed that *nod^a^* homozygotes exhibit autosomal nondisjunction when crossed to *C(2)RM* or *C(3)RM* males. Considering that *nod^a^* exhibits some autosomal nondisjunction and that *nod^143^* phenocopies *nod^a^* with respect to *X* and *4^th^* nondisjunction; this provides an explanation for the unanticipated isolation of an allele of *nod* in our screen.

Mei-218 is enigmatic in that its protein localization is predominantly cytoplasmic, yet it functions in the resolution of crossovers (Joyce *et al.* 2012; Manheim *et al.* 2002). In *mei-218* mutants, recombination is reduced to about 10% of wild type, and the crossovers that remain do not show a normal distribution (Carpenter and Sandler 1974; Manheim *et al.* 2002; McKim *et al.* 1996). The six alleles of *mei-218* isolated here exhibit a slightly stronger meiotic phenotype than do previously reported alleles of *mei-218*. The strongest *mei-218* alleles (McKim *et al.* 1996) are thought to be genetically null, as the alleles over deficiencies phenocopied the homozygotes.

**Figure 2:**
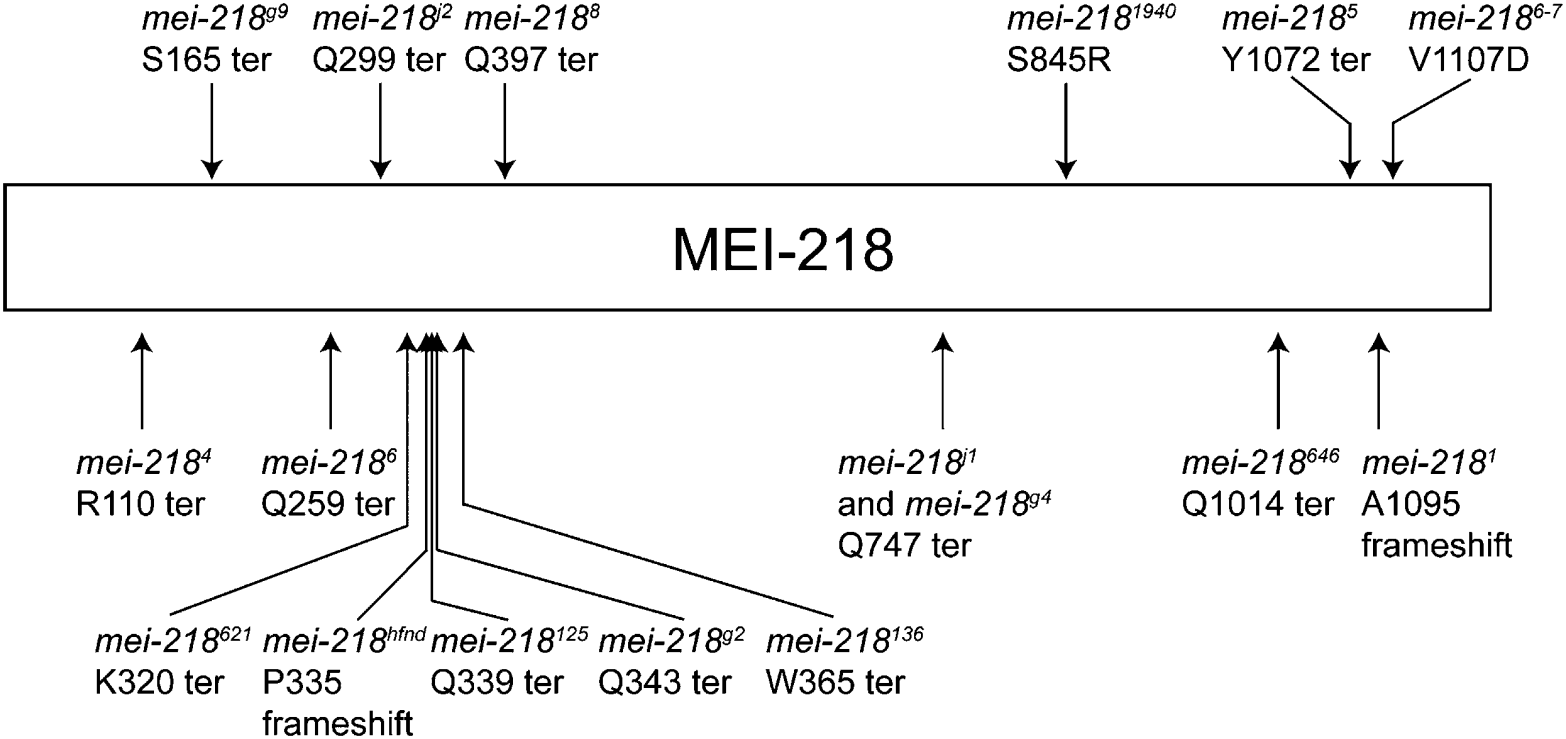
Schematic representing mutations of *mei-218* alleles. Mutations are shown for the alleles identified in this screen (with the exception of *mei-218^1057^*) as well as for the following previously identified alleles: *mei-218^4^*, *mei-218^g9^*, *mei-218^6^*, *mei-218^j2^*, *mei-218^hfnd^*, *mei-218^g2^*, *mei-218^8^*, *mei-218^j1^*, *mei-218^g4^*, *mei-218^5^*, *mei-218^1^*, and *mei-218^6-7^*. The mutation in *mei-218^7^* is not shown because it encodes for a splice acceptor mutation prior to the 4^th^ exon.

The molecular lesions of the *mei-218* alleles that we isolated in addition to other known *mei-218* alleles are shown in Figure 2 and Table 2. Notably, the mutations in *mei-218^621^*, *mei-218^hfnd^*, and *mei-218^125^* are all early terminations or frameshift mutations that occur within 17 amino acids of an 1186 amino acid protein. As *mei-218^hfnd^* is thought to be a null, this strongly suggests that *mei-218^621^* and *mei-218^125^* are also nulls that have additional mutations contributing to their stronger meiotic nondisjunction phenotype.

**Figure 3:**
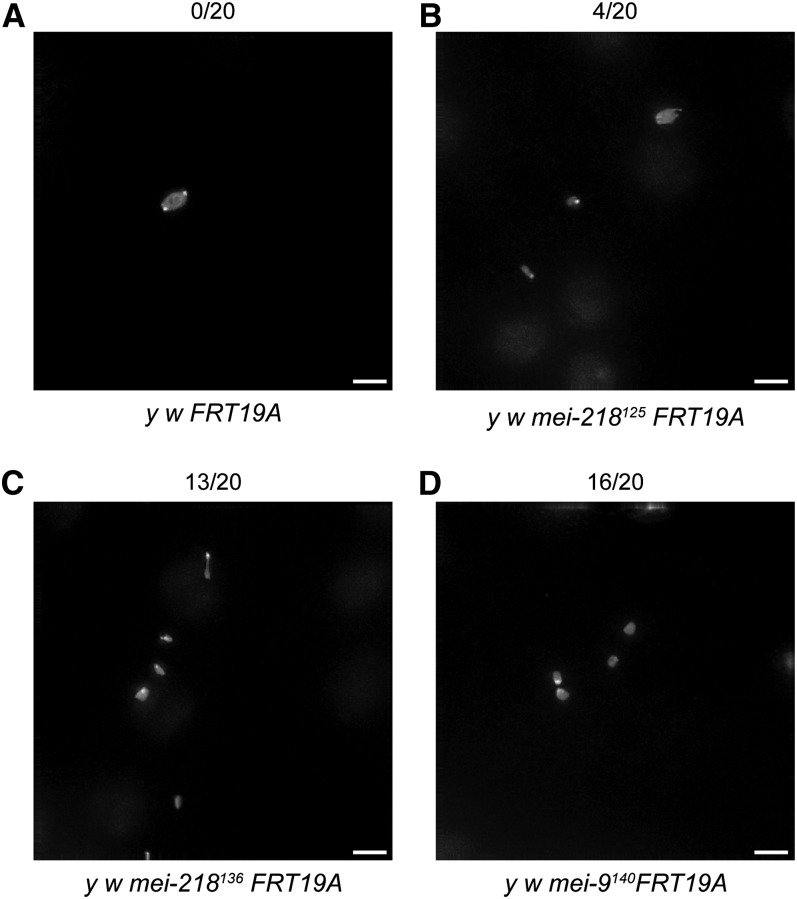
Metaphase I preparations reveal multiple chromosome masses in novel *mei-218* and *mei-9* mutants. The number of oocytes with multiple chromosome masses is indicated above each representative figure. (A) Metaphase I preparations of *y^1^ w^1118^ FRT19A* oocytes show one chromosome mass, indicating that chromosome congression is complete. (B–D) Metaphase I preparation of *y^1^ w^1118^ mei-218^125^ FRT19A* (B), *y^1^ w^1118^ mei-218^136^ FRT19A* (C), and *y^1^ w^1118^ mei-9^140^ FRT19A* (D) oocytes show multiple chromosome masses, suggestive of a defect in recombination. Scale bar: 5 μ.

Consistent with a defect in recombination, all of the new *mei-218* alleles that we analyzed exhibited multiple chromosome masses at metaphase I (Figure 3 and data not shown). In wild-type oocytes *(y^1^ w^1118^ P{ry[+t7.2]=neoFRT}19A)*, none of the 20 had multiple chromosome masses at metaphase I, indicating that chromosome congression was normal (Gilliland *et al.* 2009). In contrast, multiple chromosome masses were seen in our *mei-218* mutants. Multiple chromosome masses at metaphase I were seen in 10 out of 20 *mei-218^621^* oocytes, 11 out of 20 *mei-218^646^* oocytes, 14 out of 20 *mei-218^1057^* oocytes, 13 out of 20 *mei-218^136^* oocytes, and 4 out of 20 *mei-218^125^* oocytes (Figure 3 and data not shown). The control, *mei-218^1^*, had 3 out of 20 oocytes with multiple chromosome masses at metaphase I (data not shown). *Mei-218^1940^* was not analyzed.

*Mei-217* is expressed from the same message as *mei-218*, although they share only one part of one exon in the coding sequence. Intriguingly, *mei-217* mutants exhibit a similar recombination-defective phenotype to *mei-218* mutants. It will be interesting to localize Mei-217 to determine whether it, too, shows predominantly cytoplasmic localization. Our new allele, *mei-217^1330^*, is phenotypically similar to the two previously identified alleles of *mei-217*, called *mei-217^r1^* and *mei-217^g10^*. *Mei-217^r1^* and *mei-217^g10^* have *X* chromosome nondisjunction rates of 34.4% and 34.5%, respectively (Liu *et al.* 2000). The *mei-217^g10^* allele is thought to be a null, as *mei-217^g10^*/*Df (1)815-6* phenocopies *mei-217^g10^* homozygotes. As *mei-217^1330^* has an *X* nondisjunction frequency of 32.3%, it is likely that *mei-217^1330^* represents another *mei-217* null.

*Mei-9* is a well-characterized gene that functions in meiotic recombination and nucleotide excision repair (Sekelsky *et al.* 1995). Our two alleles of *mei-9*, (*mei-9^357^* and *mei-9^140^*) are likely hypomorphic, as they have *X* chromosome nondisjunction frequencies (21.9 and 24.2%, respectively) that are lower than the most severe *mei-9* alleles reported to date. The first allele of *mei-9* reported by Baker and Carpenter (1972) had an *X* chromosome nondisjunction frequency of 27.6%, whereas *mei-9^11^*/*mei-9^A2^* females nondisjoin *X* chromosomes 39% of the time (Yildiz *et al.* 2004). However, we have not yet assayed the *mei-9* alleles reported here over a deficiency for *mei-9* to distinguish whether our new *mei-9* alleles are hypomorphic or null. Similar to other alleles, our novel *mei-9* alleles also appear to be defective in recombination, as assayed by metaphase I chromosome preparations (Figure 3D and data not shown). Wild-type oocytes showed none out of 20 with multiple chromosome masses, whereas *mei-9^357^* and *mei-9^140^* oocytes had 13 out of 20 and 16 out of 20 with multiple chromosome masses, respectively. It will be interesting to determine whether *mei-9^357^* and *mei-9^140^* exhibit DNA damage sensitivity, a hallmark of *mei-9* mutants.

### Have we reached saturation in screening the *X* chromosome for meiotics?

To determine whether we have now reached saturation in screening the *X* chromosome for fertile meiotic mutants, we used the traditional Poisson method in which the percentage saturation of a screen is determined by 100 (1 – *e*^−^*^m^*), where *m* is defined by the average number of alleles of a given locus. Unfortunately, the Poisson distribution assumes that all genes are equally mutable (Smith *et al.* 1980). A problem inherent to this analysis is that all genes are not equally mutable: in fact, it is not uncommon for a screen to isolate many alleles of a given complementation group (Laurencon *et al.* 2004). When *m* is calculated as the average number of alleles per locus, these “hypermutable” loci lead to an increase in *m*, distorting the estimate of saturation. However, excluding what appear to be hypermutable loci at the researchers’ discretion can also be problematic.

To determine whether saturation has been reached with respect to screening for meiotic mutants on the *X* chromosome, we compiled a list of all known meiotic mutants on the *X* that were isolated from unbiased genetic screens that were fertile enough to survive a nondisjunction assay. Screens for additional alleles of a given gene were excluded from this analysis. To our knowledge, four such screens have been performed: Baker and Carpenter (1972), Liu *et al.* (2000), Sekelsky *et al.* (1999), and this article. Table 3 represents a list of all known mutations on the *X* isolated from these four screens, presented in cytological order on the *X*.

**Table 3:** Meiotic mutants on the *X* chromosome identified by genetic screens

Gene	Allele	Publication
*mei-38*	*mei-38^1^*	Baker and Carpenter (1972)
*Klp3A*	*Klp3A^352^*	Baker and Carpenter (1972)
*mei-9*	*mei-9^a^*	Baker and Carpenter (1972)
*mei-9*	*mei-9^j3^*	Liu *et al.* (2000)
*mei-9*	*mei-9^357^*	This article
*mei-9*	*mei-9^140^*	This article
*hdm*	*hdm^g6^*	Liu *et al.* (2000)
*hdm*	*hdm^g7^*	Liu *et al.* (2000)
*hdm*	*hdm^g8^*	Liu *et al.* (2000)
*mei-P26*	*mei-P26^1^*	Sekelsky *et al.* (1999)
*nod*	*nod^a^*	Baker and Carpenter (1972)
*nod*	*nod^143^*	This article
*mei-41*	*mei-41^1^*	Baker and Carpenter (1972)
*mei-217*	*mei-217^g10^*	Liu *et al.* (2000)
*mei-217*	*mei-217^r1^*	Liu *et al.* (2000)
*mei-217*	*mei-217^1330^*	This article
*mei-218*	*mei-218^1^*	Baker and Carpenter (1972)
*mei-218*	*mei-218^6-7^*	Baker and Carpenter (1972)
*mei-218*	*mei-218^j1^*	Liu *et al.* (2000)
*mei-218*	*mei-218^j2^*	Liu *et al.* (2000)
*mei-218*	*mei-218^g1^*	Liu *et al.* (2000)
*mei-218*	*mei-218^g4^*	Liu *et al.* (2000)
*mei-218*	*mei-218^g9^*	Liu *et al.* (2000)
*mei-218*	*mei-218^1057^*	This article
*mei-218*	*mei-218^646^*	This article
*mei-218*	*mei-218^136^*	This article
*mei-218*	*mei-218^1940^*	This article
*mei-218*	*mei-218^621^*	This article
*mei-218*	*mei-218^125^*	This article
*mei-39*	*mei-39^1^*	This article
*mei-39*	*mei-39^129^*	This article
*mei-39*	*mei-39^166^*	This article
*mei-826*	*mei-826*	This article

Summary of meiotic mutants on the *X* identified through unbiased genetic screens. Alleles of these genes that were identified through a targeted screen are not included in this table. *Mei-218^6-7^* is an unpublished allele from Baker and Carpenter (1972). Complementation groups are indicated by boxes.

Prior to the screen reported here, we estimated the saturation frequency of the *X* chromosome for fertile meiotics to be 87.9%; with all four screens taken together, we now estimate the saturation frequency to be 95%, resulting in a net increase of 7.1%. In all cases, *m* is calculated as the number of alleles divided by the number of loci, and percentage saturation is calculated as 100 (1 – *e*^−^*^m^*). If *mei-218* is excluded from this classical Poisson analysis (as a possible hypermutable locus), the saturation frequencies are 77.7% prior to this screen and 86.5% after this screen, resulting in a net increase of 8.8%. Regardless of whether *mei-218* is included, the *X* chromosome is reaching saturation for fertile meiotic mutants.

An alternative method relies on using a variant of the Poisson distribution in which *m* is defined based on the proportion of loci with one and two alleles, respectively. This method is ideal in the case where the proportion of loci with one and two alleles is not small, as it does not require the exclusion of data (Laurencon *et al.* 2004). However, this method is not suited to the screens of the *X* chromosome under discussion, as the number of genes defined by two alleles is equal to one when the data from all four screens are considered.

The number of fertile meiotics isolated by the Baker and Carpenter (1972) screen of 209 mutagenized chromosomes has never been matched. As shown in Table 4, six essential meiotic genes were identified in the Baker and Carpenter screen, whereas the subsequent Liu *et al.* (2000) screen isolated two meiotic genes. The Sekelsky *et al.* (1999) screen identified one critical meiotic regulator on the *X* chromosome, and the screen reported here identified two novel meiotic genes (represented by *mei-39* and *mei-826*). In total, 125,674 *X* chromosomes have been screened by these four screens alone (Table 4).

**Table 4:** *X* chromosome screens for meiotic mutants

Publication	Number of *X* Chromosomes Screened	Mutagen	Novel Genes
Baker and Carpenter (1972)	209	EMS	*nod*, *mei-218*, *mei-9*, *mei-38*, *mei-41*, *Klp3A^mei-352^*
Liu *et al.* (2000)	2,106	EMS	*mei-217*, *hdm*
Sekelsky *et al.* (1999)	2,311	P element	*mei-P26*
This article	121,048	EMS	*mei-39*, *mei-826*
Total chromosomes	125,674		

### What mutants did we fail to isolate from the screen?

As the screen required that the germline clone containing females be fertile, one limitation of the screen is that we will not recover mutants that result in a severely reduced fertility phenotype or are sterile. However, we could have recovered weak hypomorphic alleles of genes that have reduced fertility, such as *Klp3A^mei-352^*, *mei-P26*, *Cap*, and *mei-41*. One possibility for why we did not isolate such weak hypomorphic alleles is that such mutations did not cause enough autosomal nondisjunction to be picked up in the screen. Additional limitations of our screen are that maternal-effect lethals will not be identified and mutations in genes required for pre-meiotic mitoses may not be identified.

One notable gene for which we did not isolate mutants is *mei-38*. As *mei-38* mutants are very fertile (Baker and Carpenter 1972), we should have been able to isolate mutants in this gene if we were truly screening to saturation. It is possible that one of the complementation groups represented by mutant lines *mei-889*, *mei-114*, and *mei-105* is allelic to *mei-38*. However, we were unable to complete these complementation assays due to inconsistencies in the mutant homozygote phenotype.

In conclusion, the germline clone screen described here led to the identification of two novel meiotic mutants, whose characterization will be described shortly in subsequent publications. In addition to the novel complementation groups, new alleles of *mei-217*, *mei-218*, *mei-9*, and *nod* were isolated. Due to the success of this screen and the previous germline clone screens on 2*L* and 3*R* in identifying new meiotic genes (Page *et al.* 2007), it would be prudent to continue to mine chromosome arms 2*R* and 3*L* for meiotic mutants. Indeed, these screens are currently under way.
